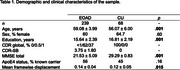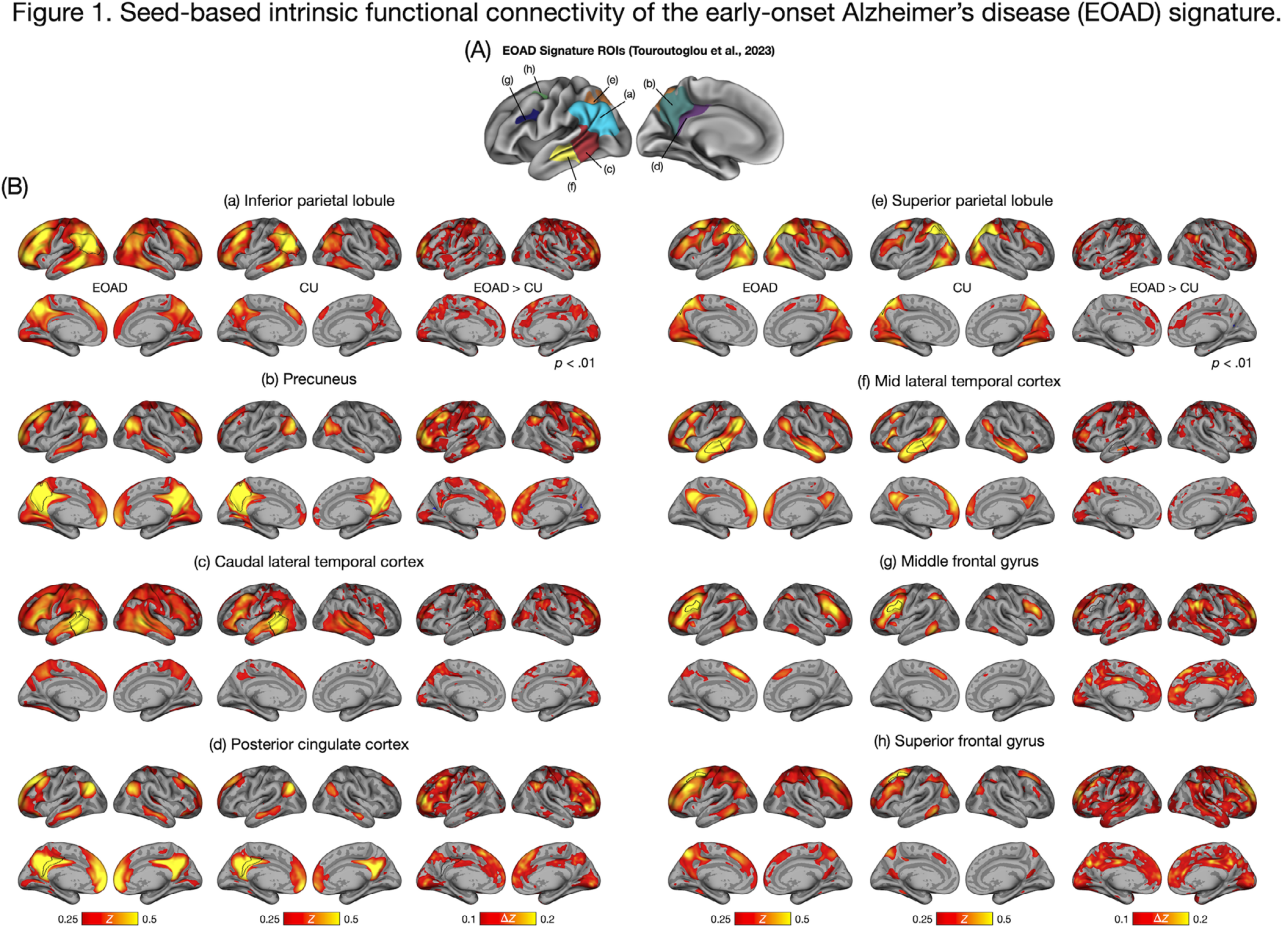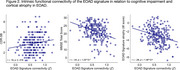# Aberrant intrinsic functional network connectivity in Early‐Onset Alzheimer’s Disease

**DOI:** 10.1002/alz70862_109821

**Published:** 2025-12-23

**Authors:** Yuta Katsumi, Ryan Eckbo, Michael Brickhouse, Ani Eloyan, Renaud La Joie, Kelly N. Nudelman, Tatiana M. Foroud, Jeffrey L. Dage, Maria C. Carrillo, Gil D. Rabinovici, Liana G. Apostolova, Alexandra Touroutoglou, Brad C. Dickerson

**Affiliations:** ^1^ Frontotemporal Disorders Unit and Massachusetts Alzheimer’s Disease Research Center, Department of Neurology, Massachusetts General Hospital and Harvard Medical School, Boston, MA USA; ^2^ Frontotemporal Disorders Unit, Department of Neurology, Massachusetts General Hospital and Harvard Medical School, Boston, MA USA; ^3^ Department of Biostatistics, Brown University, Providence, RI USA; ^4^ Memory and Aging Center, Weill Institute for Neurosciences, University of California San Francisco, San Francisco, CA USA; ^5^ Department of Medical and Molecular Genetics, Indiana University School of Medicine, Indianapolis, IN USA; ^6^ Department of Neurology, Indiana University School of Medicine, Indianapolis, IN USA; ^7^ Medical & Scientific Relations Division, Alzheimer's Association, Chicago, IL USA; ^8^ Department of Radiology and Imaging Sciences, Center for Neuroimaging, Indiana University School of Medicine, Indianapolis, IN USA

## Abstract

**Background:**

Neurodegeneration in sporadic early‐onset Alzheimer’s disease (EOAD) at the stage of MCI and mild dementia is characterized by atrophy most prominently in posterior temporoparietal cortical areas. These “EOAD signature” regions (Touroutoglou et al., 2023) spatially correspond to key nodes of several canonical large‐scale functional networks, including the default mode, frontoparietal, language, and dorsal attention networks. While current evidence points to abnormal functional connectivity in EOAD compared with healthy controls, prior studies employed small samples and have yielded mixed results. Here, we analyzed a large sample of sporadic EOAD patients from the Longitudinal Early‐Onset Alzheimer's Disease Study (LEADS) to test the central hypothesis that EOAD disrupts the functional integrity of several brain networks. We also hypothesized that AD‐related connectivity alteration would be associated with the magnitude of cognitive impairment and of cortical atrophy in the EOAD signature.

**Method:**

We analyzed whole‐brain multi‐band functional MRI data (duration = 10 min) collected at wakeful rest from sporadic EOAD (*n* = 239) and cognitively unimpaired (CU) (*n* = 68) participants from the LEADS cohort (Table 1). We used a custom pipeline to conduct preprocessing, confound removal, and quality control of MRI data. The residual functional timeseries data were used to calculate seed‐based functional connectivity of each anatomically distinct cortical region within the EOAD signature (Figure 1A). We then compared these seed‐based connectivity maps between EOAD and CU participants, while controlling for age, sex, and estimates of in‐scanner head motion.

**Result:**

EOAD patients showed abnormally stronger connectivity in all EOAD signature regions than CU participants, involving multiple large‐scale functional networks (Figure 1B). In EOAD patients, stronger functional connectivity within multiple networks was associated with worse cognitive impairment and greater atrophy in the EOAD signature (Figure 2). An exploratory analysis of whole‐cortex functional connectome further showed that AD‐related hyperconnectivity was prominently observed in relation to the default mode and frontoparietal networks.

**Conclusion:**

In patients with sporadic EOAD, phenotypically vulnerable cortical regions exhibit prominent disruption of intrinsic functional connectivity. Our findings support the selective vulnerability of cortical functional networks in EOAD, which may underlie the distinct pattern of AD pathology spreading and cognitive impairment in this AD clinical phenotype.